# Is Another kind of Biologization Possible? On Biology and the psy Sciences

**DOI:** 10.1007/s12124-023-09757-0

**Published:** 2023-03-29

**Authors:** Svend Brinkmann, Rasmus Birk, Peter Clement Lund

**Affiliations:** Department of Communication and Psychology, Aalborg University, Teglgaards Plads 1, Aalborg, 9000 Denmark

**Keywords:** Mutualism, Mentalism, Biologization, Ecology, Niches, Psy sciences

## Abstract

The relationship between biology and the psy disciplines (psychology, psychiatry, and psychotherapy) is a complex one. Many scholars have criticized how these disciplines have been biologized in the 20th century, especially since the emergence of psychopharmacology, neuroscience, and genetic research. However, biology is not just a laboratory-based science of chemical compounds, scanners, and DNA sequencing, but also a field science based on observations of organisms in their milieus. In this paper, we draw a contrast between laboratory-based biology with a focus on brains and genes, and an ecology-based biology with a focus on lives and niches. Our argument is philosophical in nature – building partly on Wittgenstein as a “philosopher of life” – to the effect that the psy sciences need not just less biologization of the former kind, but also more biologization of the latter kind to avoid a prevalent mentalism. Not least when it comes to an understanding of psychological distress, which can favorably be viewed situationally and coupled to human lives in ecological niches.

## Introduction

It is uncontroversial to claim that what Nikolas Rose (1996) has termed “the psy sciences” (psychology, psychiatry, and psychotherapy) have been much influenced by biological perspectives in recent decades. The relationship between these disciplines and biology is long and complex, including of course Wilhelm Wundt’s inspiration from German experimental physiology (see Greenwood, 2015), William James’ mind-body dualism in his *Principles of Psychology* (James, 1890) – later repudiated by James himself – and the inspiration from biology in Jean Piaget’s theory of adaptation (Piaget, 1971) to the explicit Darwinian inspiration in the development of functionalism (Greenwood, 2015), or even (much later) how figures such as Carl Rogers and Abraham Maslow explicitly drew their ideas about “self-actualization” from the work of German neurologist Kurt Goldstein (see Whitehead, 2017). Put briefly: The question of physiology and biology has presented itself to the psy-complex as both a philosophical, methodological, theoretical, and quite practical question since the early beginnings of the disciplines. Needless to say, the relationship between the psy sciences and biology has changed drastically since these early days, with significant developments such as psychiatric drug treatment, neuroimaging techniques (Spear, 2007; Rose & Abi-Rached, 2013), and behavioral genetics (Panofsky, 2014).

The triad of psychopharmacology, neuroimaging, and behavioral genetics has led some scholars to talk about a veritable biologization of the sciences and practices of the mind (Slife et al., 2010). For example, rather than working within sharp divides between ‘biology’ and ‘sociality’, or ‘nature’ and ‘nurture’, much contemporary research on (mental) health and illness today emphasizes that social life, especially so-called adverse experiences, can impact neurobiological development (e.g. in children), increasing the risk of mental health problems later in life (see Finlay et al., 2022). Such research, alongside other neurobiological findings, has significantly challenged the human and social sciences (Rose, 2013).

As shown by Fitzgerald and Callard (2015), however, some scholars have lamented the “spectre of the brain” (Fitzgerald & Callard, 2015) upon social science, while others have remained enthusiastic about its potentials, and some have suggested new paths forward for interdisciplinary collaborations. Across anthropology (e.g. Ingold & Palsson, 2013; Niewöhner & Lock, 2018), sociology (e.g. Meloni, 2014), and cultural studies (e.g. Wilson, 2004), we thus see multiple theoretical engagements with neurobiological research and the ‘materiality’ of the human body, emphasizing different paths forward for ways to rethink subjectivity and society in light of the current ‘biological age’ (Rose, 2013). Rose has argued that the human and social sciences must “move beyond description, commentary and critique, beyond the study of downstream ‘implications’ of biology and biomedicine, to develop an affirmative relation to the new ways of understanding the dynamic relations between the vital and its milieu – the vital in its milieu – the vital milieu – that are taking shape.” (Rose, 2013, pp. 23–24).

Indeed, this focus on the ‘vital’ organism in its environment serves as a focal point for our paper, which is further motivated by the fact that the discussions outlined above have largely *not* taken place *within* the psy sciences. This is especially problematic, because these trends of ‘biosocial’ research tend to reduce the psychological to an interface, which mediates between biology and society yet without making us wiser to the complexities of the psychological itself (Fletcher & Birk, 2022). In this paper, we will argue that a kind of non-reductive biologization is not only possible, but also desirable, viz. one that understands humans ecologically, as living beings in their environmental niches. Our argument will be philosophical in nature from the outset – building partly on Wittgenstein as a “philosopher of life” – to the effect that the psy sciences need not less biologization, but rather a biologization that is grounded in *ecology*, in the understanding of the ’vital’ organism in its niche. This, we find, is particularly relevant for an understanding of psychological distress, which can favorably be viewed situationally and coupled to ecological niches – or so we shall argue.

We thus ask what may happen if the biologization of the psy sciences could emanate from ecology and field biology, rather than the laboratories of pharmacologists, neuroscientists, and geneticists. The discussion will focus on three dichotomies related to the laboratory versus ecology discussion: The first is between *the neurobiological and lives* as objects of study; the second between *genes and niches*; and the third between *mental disorder as inner dysfunction* and *situational disease* (Gannik, 1995).

Three caveats should immediately be made: First, these aspects of laboratory and ecology focused research could have been contrasted in different ways. The neurobiology of brains could have been contrasted with niches, and genes with lives. So it is mainly for heuristic reasons that we have chosen to cut the cake in this particular way, but we do think that it makes sense to say that those neuroscientists who identify mental processes with brain processes could gain much from understanding them as life processes that take place in people’s ecological lifeworlds. Likewise, the ways that genes are expressed are deeply related to the niches in which humans and animals live. Second, we are unsure if the term dichotomy is really the right one here, because there is nothing to stop genetic researchers from learning from studies of niche construction – and vice versa. Indeed, this happens all the time in the science of biology, e.g., in evolutionary ecology (see Laland et al., 2016), so the two aspects are by no means mutually exclusive. We present them as dichotomies only in relation to the sciences of the mind, because we find that human scientists typically associate the term biologization with the brain/genes/dysfunction side, whereas we wish to introduce the other, and – in our view – much more promising kind of ecological biologization that focuses on lives/niches/situational disease.

Finally, and this is not so much a caveat as it is our central argument: The laboratory side of biology obviously knows that organisms live in ecologies of niches, just as the ecology side knows that organisms have brains and genes, but – to simplify a bit – the ecology side understands the laboratory side better than the laboratory side understands the ecological one. The ecological perspective understands the brain as a relational organ, for example, which mediates the relations of a living creature to the world (the brain is inseparable from a living body, which again is inseparable from its world) (Fuchs, 2018), whereas the laboratory view by necessity begins with an abstraction, e.g., the brain that is separated from its body and environment. The ecological side also knows that the laboratory itself is an ecological niche that quite specifically influences how humans act and experience the world (Martin, 2021). The laboratory side, however, has a harder time understanding the niche as ecological context. We will return to this below, but it is important to emphasize that our intention is not to falsify and reject laboratory-based research neither in biology nor in the human sciences, but rather to place it within the confines of an understanding that privileges the living organism/person in ecological contexts (niches), which may in some cases cause severe disease because of a problematic relation between organism and environment – in other words, we want to argue that the mechanism which may cause disease does not reside solely within the person or wholly outside them, but rather in the relation between the two.

## A Mutualist Biology in the Field

The contemporary biologization of the psy disciplines originates in laboratories, where scientists have synthesized and tested drugs, scanned the brains of experimental subjects, and engaged in DNA sequencing, for example. This mirrors the practices of laboratory-based biology in general that does not deal with aspects of the human mind, but with cells, tissue, organs etc. However, biology is also a field discipline, where researchers travel to forests, deserts, lakes, and seas to study the life of plants and animals. Indeed, many significant biological discoveries have been made in the field, including the revolutionary work of Charles Darwin. His study of finches in Galapagos and earthworms in England were central to his “mutualist” account of how organisms and environments are entangled and reciprocally constitute each other (an aspect of Darwinian thought that has been much overlooked; see Costall, 2004). A century later, Syunzo Kawamura and colleagues discovered in the 1950s that certain macaque monkeys on Kyushu Island in Japan teach the younger members of the troop to wash potatoes before eating them, which was also revolutionary because it falsified the idea that only humans have “culture” in the sense of knowledge that is not transmitted genetically but through education (Kawamura, 1959).

These are just a couple of central examples of how general and very significant biological principles of animal life have been articulated by researchers in the field. We may conjecture that these principles could never have been formulated if the researchers had stayed in the laboratories, studying finches, earthworms, or monkeys in cages or even in brain scanners. Qualitative observations from the field and an ecological stance were surely needed. Ecology is the study of the relationships between organisms and their environments, and an ecological stance is one that insists on observation of these relationships in situ. The mutualist principle states that organisms are both developed in and affected by their environments, but at the same time develop and affect their environments.

Since an ecological and mutualist stance will inform the arguments that follow, we will explain already at this stage what it means with a bit more detail: Anthropologist Tim Ingold has articulated the idea of mutuality with the example of the spider in a discussion with Actor-Network Theory (ANT) (Ingold, 2011). Ingold depicts a contrast between the life of the spider and the life of the ant (pun intended, as the ant represents ANT). He tells us that the ant, according to the vocabulary of ANT, acts as an act-ant (p. 90), i.e., derives its agency from the entire network of associations in which it takes part. It is a central assumption for actor-network theorists that agency is distributed across networks of actants. Furthermore, ANT scholars (e.g., Latour, 2005) will argue that anything can belong to the network, ants (or humans, for example) as well as non-ants (and non-humans), so we should in principle abstain from locating agency anywhere in particular in the network. This is a key point for actor-network theorists. Not so for the spider, however, as Ingold explains through his parable that is framed as a dialogue between the spider and the ant. For, as the very articulate spider tells the reader with reference to the web as its niche: “The web, in short, is the very condition of my agency. But it is not, in itself, an agent.” (Ingold, 2011, p. 93). The spider lives in a web – an ecological niche – that it itself has spun, and this enables the spider to catch its prey and move around in what would otherwise be free air. It is thus a creation made by the spider, and this creation simultaneously enables its life processes and constrains them. The web is a condition of the spider’s life and activities that again is contingent upon the affordances of the environment on which it was built.

Ingold’s spider thus points to a mutualism between organism and environment while still maintaining a form of organismic agency. This is inspirational for us as human scientists, who wish to study human lives in their ecological contexts, but without eliminating the agency of the subject. Although there is mutualism between organism and environment, it is still the organism that acts. Likewise, human beings live in ecological niches that are both material and social, affording various courses of action (Gibson, 1986). Our environments result from historical processes of human activity and niche construction (Sterelny, 2012). However, we still believe that it is irreducibly the human person that displays agency, intentionality, and to whom we rightly ascribe responsibility (Brinkmann, 2018).

## Why Biologize?

Before moving on to the three dichotomies that will structure our argument below, we would like to raise the question: Why advocate for a certain form of biologization instead of just joining the critique of reductive biologization? If our goal is to criticize a reductive biologization that flows from the laboratories of neuroscientists and geneticists, for example, then why not simply reject biologization as such? Why do we put forward the question whether another kind of biologization is possible?

The short answer is that we find that much research from within the psy sciences is inadequate on its own terms. It is not possible to provide a thorough analysis here, but our view is that a common understanding of human beings in psychology is based on individualist and representationalist perspectives. This means that thoughts and feelings are pictured as processes that take place within an individual’s mind, and they operate by way of mental representations of objects and events in the outer world. This basic image of the human being may be called *mentalism* and is rooted in a philosophical tradition that goes back at least to Descartes in the 17th century. Descartes believed that the mind was a thinking thing – an independently existing metaphysical realm – which was connected to a living organism almost only by accident. For Descartes the mind was a substance, a kind of medium for the metaphysical soul, and the body was simply a dead, mechanical object.

Although there are practically no psychologists and philosophers who adhere to Descartes’ form of mentalism today, mentalism has nevertheless survived in many newer guises and is often found, paradoxically enough, among researchers who explicitly criticize Descartes – certain materialists, for example, who believe that everything in existence must be understood in terms of physical matter. Some of them can be criticized for being “neural Cartesians”, since they may well have rejected Descartes’ idea of the mind as an independent metaphysical substance, but at the same time they have retained his idea that the psychological realm is an inner, private world of mental representations, which is now merely believed to exist in the brain and not as a substance of the soul (Coulter & Sharrock, 2007, p. 11).

In the 20th century, mentalism came under attack by some of the most significant philosophers of the time, including Ludwig Wittgenstein, and also a number of phenomenologists such as Heidegger and Merleau-Ponty, whom we will not address in this context. They problematized the key idea of mental representations (see Brinkmann, 2022). For if such representations are supposed to explain how we can recognize something as being a certain thing, we basically duplicate a problem by creating a fiction. Simply put, the representational theory claims that the mind can recognize a beech tree as a beech tree, because the knower has formed a mental representation of beech trees. A reference to mental representations does not solve this problem, however, but merely serves to transform it from a problem about mind and world to a problem about mind and inner representation. For we can then continue to ask how the person can know that the inner, mental representation depicts a beech tree? If it is difficult to explain how we can recognize an object in the outer world as a beech tree, why is it thought to be easier to double the problem and try to explain how we can recognize that the inner mental representation depicts a beech tree? This does not solve any problems; it merely creates new ones. Mentalism simply moves the problem into our heads.

Wittgenstein (1953) would insist that a private mental representation can never have meaning, because meaning is connected to use in public social practice. Mentalist talk of inner representations makes the problem unsolvable, because mental representations cannot mean anything in and of themselves. The problem arises from the Cartesian distinction between mind and matter that gives rise to the unsolvable question: How can the alleged contents of a mind represent states of affair in the material world outside? So, instead of beginning with this dead end, Wittgenstein argued that we should begin by acknowledging the division between the living and non-living as more basic and important. Representationalist *mind-talk* should thus be replaced by vitalist *life-talk*, *mentalism* should be replaced by *mutualism*, and we believe that this would represent a giant leap forward for the psy sciences that has not yet been properly explored (Brinkmann, 2020). In a helpful exposition of Wittgenstein’s influential book, *The Philosophical Investigations*, McGinn (1997) unfolds its major themes, centered on Wittgenstein as a philosopher of life: “the emphasis on the body as the objectification of the human soul; the replacing of the division between matter and mind with the division between the living and the non-living” (p. 8). This is where Wittgenstein’s ideas are relevant for a non-reductive biological view of life.

Wittgenstein was what we may call a philosopher of life in the Aristotelian sense (see Brinkmann, 2020, on which some of the following build), who saw “Commanding, questioning, recounting, chatting […] as much a part of our natural history as walking, eating, drinking, playing.” (Wittgenstein, 1953, § 25). Wittgenstein saw his own project as one of providing remarks on “the natural history of human beings” (§ 415). What we can call communicative practices – writing, gesturing, speaking, shouting, whispering, and so on – are quite simply the *nature* of human beings.

A key term for a Wittgensteinian natural history of human beings is *form of life*: “to imagine a language means to imagine a form of life” is one of the most famous remarks from the *Philosophical Investigations* (1953, § 19). Form of life, of course, should not be understood in strictly biological terms. It refers, rather, to how human beings live, grow, develop together and how these processes are both biological – we breathe the air, eat, drink, etc. – but also always bound to cultural practices. Eating, drinking etc. are deeply entwined with cultural practices, beliefs, rituals. Human development is not about the internalization of a ‘mind’, but about the embodied development within such forms of life. As McGinn puts it:

The structure and complexity of psychological states does not lie in the structure and complexity of some inner mechanism (mental or physical), but in the complex (temporally extended) form of life that is apparent in an individual’s behavior and accessible from a third-person point of view. (McGinn, 1997, p. 56).

The human sciences should thus study temporally extended forms of life instead of inner representations and mechanisms. Understanding the words and practices of one’s culture, for example, is not linked to mental representations “in the mind” of the individual, but rather to the person’s ability to participate with others and go on according to the norms of the shared practices. Wittgenstein claimed that that “the human body is the best picture of the human soul” (Wittgenstein, 1953, p. 178), an idea close to the ideas of, for example, Aristotle (Ostenfeld, 2018, p.67), who saw the soul (psyche) as a principle of life.

The body’s liveliness, McGinn argues, consists “not merely in the sense that it moves about, but in the sense that the continual play of its movements and gestures have a particular meaning or significance.” (1997, p. 155). There is no need for an inner, mentalist realm. Intentions are perceivable – in a way like what is pointed out in the work of James J. Gibson, to whom we return. We do not need to conduct a series of mental calculations to infer what a particular gesture *means*. Instead, the gesture *is* the meaning (within a cultural, political, social, historical context) and this is immanent to the gesture. Wittgenstein illustrates this with examples from other species. We should look to a cat, he writes (Wittgenstein, 1953, § 647), as it stalks its prey. Its intentions do not lie in any inner mental representations, but rather lies within the behavior, in the movements themselves. Life, in this sense, manifests itself. In this way, Wittgenstein prefigured the later interest in embodiment across the human and life sciences (see Krieger, 2005). The psy sciences should from this perspective be understood as sciences of *living* creatures and of how these creatures conduct their *lives* in environments that should be characterized in *mutualist* terms.

This brief excursus through the philosophy of Wittgenstein is simply meant as an indication why we believe that psychology and related disciplines need to move away from mentalist models. Instead of focusing on the individual, representational mind, we need a notion of living, moving, acting, thinking, feeling human beings. Our reading of Wittgenstein as a philosopher of life aligns his work with the phenomenologies of Heidegger and Merleau-Ponty (see Gier, 1981), on which contemporary scholars like Ingold (2011) build, who wish to begin a human science not with a disembodied mind, but rather with a living creature in its milieu (we return to this below). Given these perspectives, the psy sciences need to learn from biology as the science of the living, of living organisms in their ecological niches, albeit in the proper non-reductive way. We will now explore three lessons to learn based on three dichotomies.

## Neurobiology and Lives

The first dichotomy that we will focus on is that between neurobiology and lives. By *neurobiology* we mean here both the study of the brain, and the many other complex parts of physiology that are often examined by the psy disciplines, such as hormones and other biomarkers. By *lives*, as we will detail below, we mean the on-going ‘lived-ness’ that human being entails, the *conduct* of life by beings embedded within moral, cultural, and material worlds. Our argument here is *not* that research which focuses on the brain, neurobiology, hormones or similar phenomena is irrelevant, but rather that the point of departure for the psy disciplines ought to be lives, both in the sense of organisms “being alive” (Ingold, 2011), but also in the sense of people “conducting a life”, i.e., living life as a story or connecting thread that runs from birth to death (Brinkmann, 2020).

No doubt, some might find this controversial. The brain, they might argue, is an immensely complex organ and undoubtedly a hugely influential part of being human, a central and constitutive part of those complexities we call ‘emotions’, ‘behavior’, ‘feelings’, ‘thoughts’ and so on. So, too, is neurobiology more broadly understood: We know that the nervous systems, neurotransmitters, hormones and much else is deeply involved in and central to human behavior. For example, it is well-known that when human beings find themselves in so-called stressful situations, a complex neurobiological process unfolds, involving both the brain and nervous system, the hypothalamic-pituitary-adrenal axis, releasing both neurotransmitters and hormones to mobilize the organism’s resources (see Birk, 2021). Here, we thus see how contemporary theories of stress and depression take their departure in detailed examinations of our complex neurobiology.

Given the scientific findings concerning both the chemistry and structure of the brain, however, it is relevant to remember what the brain actually does when it shapes and determines our body’s functions – biological as well as psychological. Simply put, the brain’s function, as an organ, is basically to send electro-chemical signals along specific circuits, which are necessary for our organisms to be able to live, breathe, keep balance, move and so on. Our biological and psychological functions are thus dependent on cerebral processes. Without brain processes we could not think, feel, or act. That is a simple truism. But does that mean that mental functions are *in* the brain or, for that matter, in our hormones? Does it mean that thoughts and feelings are “inside the head”? Does it mean that it is the *brain* that thinks, feels and acts?

No, regardless of what empirical neuroscience might teach us, it is a mistake to attribute abilities and dispositions to the brain, which only make sense when attributed to the living organism as a whole. Many people – also esteemed scientists – make this mistake, which has been dubbed “the mereological fallacy” by scholar working in the tradition of Wittgenstein (Bennett & Hacker, 2003). Mereology is the study of the relationships between parts and wholes, and the mereological fallacy consists in attributing characteristics to a part of something (e.g., the brain), where it is only meaningfully attributable to the whole (e.g., the living organism). The famous neuroscientist Antonio Damasio, for example, commits a mereological fallacy when he describes how the brain is aware of an image of an object, or when he analyses how the brain perceives something (Damasio, 1994, p. 263; 1999, p. 189). However, the brain *itself* cannot be aware of anything or perceive anything. What it can do is transform energy, and this energy transformation obviously affects how a *person* can be aware of something or perceive something during that person’s life, so this does not change the fact that it is the living person and not the brain that can do these things.

The mereological fallacy is widespread. To mention just a couple of additional examples: Francis Crick writes that the brain has “convictions” and that it can “guess” and “interpret”; Gerald Edelman argues that the brain “categorizes” and “manipulates the rules”, Colin Blakemore writes that neurons have “intelligence” and “knowledge” and that they “present arguments” for the brain (Bennett & Hacker, 2003, p. 68), while newer theories posit that the brain is “predictive” or even “Bayesian”, making inferences (or predictions) about its environment (Friston, 2012). Importantly, if it does not make sense to attribute psychological characteristics (paying attention, for example) to the brain, then it does not make sense to attribute their *negations* to the brain either. The fact that the brain cannot pay attention does not mean that it is therefore inattentive. The fact that the brain cannot see does not mean that it is therefore blind – in the same way as the oatmeal we had for breakfast cannot be said to be either awake or sleeping! This is not an empirical fact about brains and oatmeal, because we cannot someday *discover* that the brain can actually see, or that our oatmeal can actually sleep. Rather, it is a conceptual fact, i.e., a fact about how we can meaningfully use the psychological terms of our language. Wittgenstein expressed this point as follows: ”…only of a living human being and what resembles (behaves like) a living human being can one say it has sensations; it sees: is blind; hears; is deaf; is conscious or unconscious.” (Wittgenstein, 1953, § 281). Our mental concepts can only be applied to living creatures with a certain range of behaviors – and the brain is not a creature in the required sense. It is an organ of such creatures.

So even though the brain is an amazing and fascinating organ, it is still an organ – and nothing else. Fuchs (2018) has also diagnosed numerous instances of the mereological fallacy in the neurosciences that often spill over into reductionistic models in psy sciences such as psychology and psychiatry, leading us to forget that it is persons – and never their brains – that think, feel, and act. And Fuchs argues that brain states do not “describe the world” or have “representational contents”, but rather “*participate* in the functional cycles from which those contents result.” (p. 133). The brain, according to Fuchs, is an organ of the body that mediates the many ways in which living persons are related to the world. Such relations are not representational, but connected to actions, practices, and experiences. Similarly, in the case of the hormonal cascades that constitute the neurobiology of stress, it is crucial to recognize that the ‘stress process’ is widely recognized as being concerned with the *perception* of something as a *stressor* or *threat* to the organism. This means, as argued by Birk (2021), that the stress process is not, and cannot be, the value-free perception of ‘objective’ stressors: the stress process is bound to the mutuality between organism and environment. A bicyclist is rarely perceived as a threat by a human being strolling down the street but may well be perceived as such by the small dog they are walking. The perception of the threat and nonthreat is bound to the *form of life* of the organism. This means that *beginning* with the brain, or neuro-hormonal cascades, in trying to understand humans, is fallacious. It is *the unfolding life process* of an organism that is the relevant unit of analysis, and neurobiology *mediates* the capabilities and life functions of this organism.

If so, we must consider the idea of life as basic and think of the psy disciplines as *sciences of life* (Brinkmann, 2020). This argument has been made by Ingold specifically in relation to anthropology, but we believe it applies more generally to the psy disciplines as well. In Ingold’s aptly-titled book *Being Alive*, where he refers to his project as one of “restoring life to anthropology” (2011, p. 14), he sees his project in continuation of the ecological psychology of James Gibson and the phenomenology of Maurice Merleau-Ponty, whose approach (Merleau-Ponty’s) Ingold characterizes as follows: “his conclusion was that since the living body is primordially and irrevocably stiched into the fabric of the world, our perception of the world is no more, and no less, than the world´s perception of itself – in and through us.” (p. 12). Life is the way that the world relates to itself, and in the case of humans this happens in a reflective way. From Ingold’s perspective, there is no mind standing opposed to a world that is to be known and controlled, but only a world that perceives itself through various life processes or (as Wittgenstein would say) forms of life. This can happen reflectively in the case of humans, but not even here is knowing a matter of creating correspondence between the “contents of the mind” or “mental representations” and “the world out there”. Rather, as Ingold puts it:

knowing does not lie in the establishment of a correspondence between the world and its representation, but is rather immanent in *the life* and consciousness of the knower as it unfolds within the field of practice set up through his or her presence as a being-in-the-world. (Ingold, 2011, p. 159; italics added)

We should begin, not from the brain in isolation or from a knowing mind, but from *the life of the knower*. These knowers are, as persons, not discrete entities, containers, or vessels of representations, beliefs and desires, but rather life *movements*:

persons are not beings that move, they *are* their movements. It is in their very patterns of activity that their presence lies. And places are not so much locations to be connected as formations that arise within the process of movement, like eddies in a river current. In short, in such a world names are not nouns but verbs: each one describes a going on. (p. 168)

“To human is a verb”, as Ingold insists in a related text (2015, p. 115). In order “to human”, we might say, a human being is not just alive in a physiological sense but conducts a life by exercising its capabilities. This is where life becomes more than a brute biological occurrence (what the Greeks called *zoe*), viz., life as *bios* in the sense of a story of interconnected human actions that can be told as a *biography* (see Arendt, 1958) – literally a life written down.

From similar reflections, Fuchs (2021) concludes that it is not the brain that executes the various life functions of living human beings, but it is rather these functions that primarily affect the workings of the brain. The brain is surely an organ that mediates life processes but is not itself an agent. Fuchs expresses this as follows: “it is not the brain that generates the function, but conversely *the function, in its embodied execution, that creates its cerebral organ*.” (p. 162; italics in the original). This explains why the plastic brain can often be healed, even after serious lesions, by the living patient, who practices the life functions.

To sum up, a biologization that focuses only on neurobiology is reductive and fallacious. A mentalist focus on inner representations is not much better for reasons laid out above. Instead, we propose to focus on the irreducibly *living persons* who think, feel, and act, and do so as part of their life processes. Psychological phenomena are displayed and conducted as part of a *biography*, and it is thus the person’s *life* that is the proper object of study for the psy sciences, which turn out to be life sciences in a non-reductive sense (Brinkmann, 2020). Crucially, this does *not* mean that for example the brain can no longer be studied or that hormones should not be considered in psychological research. Rather, it specifies a point of departure: Rather than beginning with the neurobiology, in all its complexities, we should begin with *lives*; lives which are “lived through a body (not only through cells) and as a society (not only as a species)” (Fassin, 2009, p. 48). The question about how society, as an overarching structure of collectives and their practices, is connected to human lives in ecosocial niches is beyond the scope of this paper, but something to which we would like to return in a later paper. The ecological perspective, e.g., of Gibson, whom we will treat below, has been criticized as inadequate for an understanding of larger societal processes and the discussion can likely be informed by including cultural-historical approaches to human societies (e.g., Mammen & Mironenko, 2015).

## Genes and Niches

The next dichotomy is that between genes and niches. Our point here, following the focus on *life*, is that the psy disciplines should orient themselves towards *niches* first, and genes second. The niche, Gibson once argued, referred to “*how* an animal lives [rather] than to *where* it lives.” (Gibson, 1986, p. 120; emphasis in original). If the psy disciplines should focus on *lives* before neurobiology, then accordingly they should also focus on *niches* – how humans live – before DNA and genes. Before developing this argument further, we need to examine how the psy disciplines have been perhaps most oriented towards the study of genetics, namely in *evolutionary psychology*. This is a contentious field, built upon a series of deeply questionable propositions and assumptions about human nature. While few would argue that evolution has played no part in the shaping of human beings, evolutionary psychology proceeds from a particularly problematic premise, entailing (at least) the following ideas:

1. Human minds consist of numerous “information-processing” modules which have evolved because they have solved adaptive problems.
2. These modules evolved in early in human history (100.000 years ago or more – it is rarely specified), *hence* they are adapted to radically different environments and challenges than humans face today (see Cosmides & Tooby, 2005; Confer et al., 2010; Smith, 2020).

There are numerous critiques of and controversy over evolutionary psychology. For example, Subrena E. Smith has argued at length that evolutionary psychology faces an insurmountable problem. It proposes explicitly that human beings “contain” psychological adaptations that “operate today as they did in the past” (Smith, 2020, p. 41). However, this means that evolutionary psychology has a “matching problem”, for how do we know that adaptations, which can supposedly be inferred *today*, solved the same challenges (and are indeed the same adaptations) as were the case when they first evolved? As Smith puts it, the significant methodological challenge is to determine that the “function of a contemporary module is one that an ancestral module was selected for performing” (Smith, 2020, p. 42) *and* to determine that our “contemporary modules” have functions *because* they are descended from these “ancestral modules” (see Smith, 2020). Elsewhere, Ingold has argued that the evolutionary psychologists misunderstand natural selection: “Natural selection, in short, may occur within evolution, but does not explain it.” (2000, p. 243). Why not? Because the idea of the genotype, which is necessary to account for the link between gene frequencies and capacities of organisms that are independent of the concrete dynamics of development, is dubious (p. 243). According to Ingold, there simply is no such thing as a genotype conceived as a context-independent design specification (p. 234). It is impossible to factor out what the purely genetically based capacities of humans are (that are thought of, by a number of evolutionary psychologists and behavioral geneticists, as identical in the Pleistocene and today), and what is ontogenetically acquired. There are no pre-programmed essences in the genes (see Brinkmann, 2011).

Again, just as it was necessary to understand what the brain is as an organ in order to put it in its right scientific place, so it is with genes. Genes are parts of long molecules (DNA) that control the production of proteins in organisms, but genes only interact with cells that are the building blocks of bodies that operate in environments. Natural selection thus works on organisms in environments, and not on genes. And what organisms are is a result of complex developmental processes that cannot be predicted by looking solely at genes. Evolution, according to Ingold’s more complex and dynamic view, “is the process in which organisms come into being with their particular forms and capacities and, through their environmentally situated actions, establish the development for their successors.” (Ingold, 1998, p. 95). Natural selection may play a role in this process, and it would be foolish to deny that cumulative changes in gene frequencies within populations take place over time, but Ingold’s point is that there is no link between change in gene frequencies and the capacities of organisms independently of the dynamics of development itself (Ingold, 2000, p. 243). The evolutionary-developmental process is primary as such and includes both what we conventionally refer to as natural and cultural aspects – in other words: the ecosocial niche in which organisms live. Natural selection does not “design” organisms, as evolutionary psychologists say, and the role of genes cannot be specified independently of other factors (Derksen, 2010, p. 480; see also Stotz, 2014, on extended evolutionary psychology).

Human scientists should resist treating the organism and environment as two separate entities, with the environment existing prior to the animal/person (Withagen & van Wermeskerken, 2010, p. 495). This is problematic for the plain, yet radical, reason that there simply is no environment independently of organisms. There certainly is a physical world, but this world becomes an environment (with selection pressures) only when there are animals that operate in it and determine what constitutes the environment through niche construction. Even the lowliest animals, like the earthworms analyzed by Darwin himself, construct their environments (see Costall, 2004). Or, to reiterate the dynamic term introduced above, there is a *mutualism* at work in which animals and environments construct each other. The earthworms, which are actually not well adapted to life in the topsoil due to their sensitive epidermis that must be kept warm and moist, create vegetable mold in their life processes and thereby change the topsoil in which they live (Withagen & van Wermeskerken, 2010, p. 499). Even such primitive animals construct the very environments in which selection takes place, thus complicating the simple model of natural selection working on genes. The offspring of the earthworms inherit not only genes, but also an environment that fits their epidermis better (p. 500). Have birds developed color vision because apples are red, thus enabling them to locate and eat the nourishing fruits, or have apples developed their red hue because birds have color vision, thereby attracting the birds to help spread the apple seeds? Obviously, both are correct, and the mutualism works even more pervasively in the case of humans with their immense niche construction capabilities, which involve complex forms of semiosis and cultural life.

## Disorder as Essential Dysfunction and as Situational

Obviously, many people are interested in the psy sciences not just because of basic research into the minds and lives of humans, but because of a wish to help others in distress. Thus, psychiatrists, psychologists, and psychotherapists work on mental illness and disorder. But how should mental disorder be understood? With this question, we arrive at our final dichotomy, depicting a contrast between an essentialist position that is often coupled with perspectives on dysfunctional brains and genes and a position that views illness as situated in ecosocial niches.

The term used to characterize biologization with regard to mental disorder is “biomedicalization” (Clarke & Shim, 2010). Despite the fact that no simple biomarkers have been found for any mental disorder in psychiatry (Singh & Rose, 2009)^1^, and even though psychiatric diagnoses can only be formulated by evaluating and counting symptoms (and not through brain scans or blood tests), there is a huge interest among researchers in the neuroscience of mental disorder that trickles down to the public and its “folk psychiatry” (Brinkmann, 2016). Again, our argument is not that the brain is unimportant in relation to mental disorders (quite the contrary), but simply to point out that it is not possible to diagnose any mental disorder by looking at a person’s brain. The enormous interest in the neurosciences of mental disorder in recent years (depicted and analyzed in Rose & Abi-Rached, 2013) goes hand in hand with the success of *Big Pharma*, if not concerning the treatment of mental disorder (where the results are mixed), but rather concerning the marketing and spread of pharmaceuticals against a number of conditions such as anxiety, depression, and ADHD. Today, the pharmaceutical industry is among the largest in the world with marketing budgets that are twice as large as the money they spend on research and development of new drugs (Frances, 2013).

It seems problematic for anyone subscribing to an essentialist model of psychopathology that no “essences” (in the sense of simple biomarkers) have been found in psychiatry. Essentialism rests on what the historian of medicine Charles Rosenberg has called disease specificity. This which refers to the idea that diseases are specific entities that have a kind of independent existence beyond their unique manifestations in sick individuals (Rosenberg, 2007, p. 13). Rosenberg’s point is that before the 19th century, there were primarily sick people, and after there are now actual diseases. Although the idea of disease specificity might apply to somatic medicine, where examples such as cancer or diabetes can serve as obvious illustrations, it seems quite problematic in psychiatry, where Frances has reminded us that “Billions of research dollars have failed to produce convincing evidence that any mental disorder is a discrete disease entity with a unitary cause.“ (Frances, 2013, p. 19).

Furthermore, and possibly even more detrimental to essentialism, is the sociological insight that disorders “are only intelligible due to the normative and social context in which they are found” as Bowden has argued (Bowden, 2014, p. 422). Among philosophers of psychiatry today, there is widespread agreement that one cannot define anything as a disorder in the absence of social norms of normality and suffering (Horwitz & Wakefield, 2007). Even if we grant that what is today referred to as mental disorders represent ubiquitous traits in human populations across time and place (as some evolutionary psychologists will say, see Bolton, 2008), we still need social norms in order to establish whether this trait represents a disorder or simply is “a way of being human”. Such arguments have led some to the belief that mental disorders are nothing more and nothing less than contingent social constructions. This, obviously, is the anti-thesis to essentialist accounts, which is often referred to as social constructionism. Social constructionist models of disorder dissolve essentialist definitions and claim that the whole range of problematic human behaviors represent disorders only because of social categorizations and not because of anything inherent in the people who suffer. Social constructionist models come in many versions that are more or less epistemologically and metaphysically radical, but they all seem to “encourage the view that social values and priorities are the sole historical determinants of medicine, music, and marriage”, as Church writes in her account of social constructionist approaches to mental disorder (Church, 2004, p. 393). In other words, for a social constructionist, there are no mental disorders “out there”; they are all in the eyes of the pathologizing beholder.

This is not a view that we wish to endorse. Fortunately, we do not have to choose between a strict essentialism on the one hand and a radical social constructionism on the other. A properly biologized approach to distress represents a middle-ground that at once acknowledges a role for bodies, brains, genes, and other material factors on the one hand and also understands the necessity of viewing disorder as related to ecosocial niches on the other. The Danish medical sociologist Dorte Gannik devoted much of her career to developing a situational theory of illness, which is deeply congenial to our approach in this article (Gannik, 1995, 2005). Her work, which was based on studies of somatic illness, notably back problems, is little known outside Scandinavia, but her theory is noteworthy because of its simple elegance. While it is certainly relevant in relation to somatic medicine, it seems to be even more to the point concerning psychiatric problems. Gannik rejected essentialist theories of illness and disease (although there is a conventional distinction between illness and disease, this is rendered problematic by her theory). She saw illness as something relational, “identical with a person’s interactive relationship with her surroundings”, and also as performative: “The theory abstains from approaching illness as something ‘in itself’ beyond those actions or reactions with which a person responds to everyday, bodily experiences” (p. 332). She refers to the well-established fact that symptoms are pervasive in our lives, and most of us experience unpleasant sensations in our bodies every day. But symptoms are not diseases or disorders. She argues that we should talk about illness, disease, or disorder as something people “do” (perform, enact) in relation to physical and social environments. They exist in and through the ways in which they are performed as life processes within (what we call) ecological niches.

A related approach has been articulated by ethnographer and philosopher Annemarie Mol, e.g., in her study of artherosclerosis (Mol, 2002). Mol says that we should not think of diseases as “constructed”, since this metaphor solely emphasizes human symbolic activities of social construction, thereby leaving out the body and the material world (p. 32). Instead, we should use the metaphor of performance or talk about *enactment*. Referring to our discussion above, we will suggest the term *life* once more and argue that disease is something “lived”. Objects such as disorders are lived or enacted in practices, but this should not lead us to the view that they are simply done by discrete actors (who could just choose to do otherwise). Instead, the idea should suggest “that activities take place – but leaves the actors vague.” (p. 33). Enactments presuppose a whole range of mediators that make the doing possible. If we ask: “Who does the doing?” (e.g., of ADHD, OCD, anxiety etc.), then the answer has to include not just the suffering person, for events “are made to happen by several people and lots of things. Words participate, too. Paperwork. Rooms, buildings. The insurance system” (p. 25). The complexity involved leads to a need for what Mol calls a “praxiographic appreciation of reality” (p. 53), which studies how things are brought into being in socio-material practices. Contra essentialism, this means that it becomes impossible to isolate the “essence” of a disorder in any one place (e.g., the brain), and, contra social constructionism, it means that many forces besides the purely symbolic or discursive are involved. Again, we find ourselves with a mutualist position that integrates the person and the environment, the organism and the niches.

Returning to Gannik we can say that her model likewise does not isolate illness/disorder to anything in the person as such (essentialism), nor to anything in the social system in itself (constructionism). Rather, illness and disorder are always found in a relation between a person (or organism) and life situations (constituted by socio-material practices). This relational perspective means that people’s problems are always situated. They exist in their concretely lived and situated manifestations only, and not in anything behind or beyond this. Thus, essentialism fails. But it also means that the problems are irreducibly real – as real as anything gets – and sometimes stubbornly real. Thus, social constructionism in its radical versions fails. We should maintain a moderate social constructionist outlook, because certain important factors related to mental disorder are socially constructed (e.g., some norms inherent in our social practices), but we should also acknowledge the importance of factors that cannot be said to be socially constructed. It is the relation between these factors that should be in focus. Gannik’s theory implies that treating people’s disorders relationally may involve changing the person (e.g., through cognitive techniques or drugs) or changing the socio-material practices (e.g., by transforming the environment, inventing new cultural prosthetic devices etc.). The point is that someone’s problems are not to be located in a single place but dispersed over multiple mediators that unfold in the course of living.

Without referring to Gannik, the psychiatrist Fuchs has developed a similar call for psychiatry as “relational medicine”. It builds on an “ecological concept of the psyche as the overarching form of the relations between organism and environment, between person and world” (2021, p. 190). And elsewhere Fuchs calls for:

A biological psychiatry in the appropriate sense would therefore need an adequate notion of the *biological*, namely as the life process connected with the entire organism and its interactions with the environment. It needs an ecological theory of biology that integrates the social and biological processes outside the brain, even though these are functionally sedimented in genome and brain structures. (Fuchs, 2018, p. 276).

## Conclusions

We ended with a quote from Fuchs, which contain many of the necessary ingredients for a non-reductive biologization of the psy disciplines that we are looking for. Life processes are placed at the center of attention, but within an ecological perspective that does not ignore the importance of the brain and genes, but rather understands these as functionally important mediators for life processes. We believe that research that operates along these lines is what is now needed in psychology, psychiatry, and psychotherapy. It is research that will avoid the dead ends of a mentalism that locates the mind in the head of the individual (in the form of mental representations), and tends to intervene mainly, or only, at the level of what takes places “inside” the person.

The alternative to mentalism, we have argued, is not a reductive biologization that finds inspiration in the laboratories of neurobiologists, geneticists, or psychopharmacologists. Instead, we need a non-reductive biologization that approaches the psy sciences as “sciences of life” and posits the lives of human beings as the relevant unit of analysis and intervention. This will involve a focus on life processes in both physiological and biographical senses, and an awareness of the niches in which life processes unfold and which are co-constituted by living human beings. When things go badly in human lives, we should think in terms not simply of essentialist dysfunctions, but in terms of situational disease and distress centered on a relation between the living person and the environment. In short, we need mutualist psy sciences that learn from those biologists who study living organisms ecologically in their niches.

## Footnotes

1 A recent review of the search for biomarkers in psychiatry concluded: ”As set out in this review, there are several proteins, metabolites and genes that have been linked with certain neuropsychiatric diseases mainly due to the advance in ‘omics’ technologies. However, none of them have demonstrated to be a real and useful biomarker in clinical practice.” (García-Gutiérrez et al., 2020).

## References

[CR1] Arendt, H. (1958). *The Human Condition* (This edition 1998). Chicago: University of Chicago Press.

[CR2] Bennett MR, Hacker PMS (2003). Philosophical foundations of Neuroscience.

[CR3] Birk RH (2021). On stress and subjectivity. Theory & Psychology.

[CR4] Bolton D (2008). What is Mental Disorder? An essay in Philosophy, Science, and values.

[CR5] Bowden G (2014). The merit of sociological accounts of disorder: The attention-deficit hyperactivity disorder case. Health.

[CR6] Brinkmann S (2011). Can we save Darwin from evolutionary psychology?. Nordic Psychology.

[CR7] Brinkmann S (2016). Diagnostic cultures: A Cultural Approach to the pathologization of Modern Life.

[CR8] Brinkmann S (2018). Persons and their minds: Towards an integrative theory of the mediated mind.

[CR9] Brinkmann S (2020). Psychology as a science of life. Theory & Psychology.

[CR10] Brinkmann S, Slife B, Yanchar S, Richardson F (2022). Minds, brains, or persons? What is psychology about?. Routledge International Handbook of theoretical and philosophical psychology: Critiques, problems, and Alternatives to Psychological Ideas.

[CR11] Church J, Radden J (2004). Social constructionist models: Making order out of disorder - on the social construction of madness. The Philosophy of Psychiatry.

[CR12] Clarke A, Shim J, Pescosolido BA, Martin JK, McLeod JD, Rogers A (2010). Medicalization and biomedicalization revisited: Technoscience and transformations of health, illness and american medicine. Handbook of the sociology of Health, illness, and Healing.

[CR13] Confer JC, Easton JA, Fleischman DS, Goetz CD, Lewis DMG, Perilloux C, Buss DM (2010). Evolutionary psychology: Controversies, questions, prospects, and limitations. American Psychologist.

[CR14] Cosmides L, Tooby J, Buss DM (2005). Conceptual foundations of evolutionary psychology. The handbook of Evolutionary psychology.

[CR15] Costall A (2004). From Darwin to Watson (and cognitivism) and back again: The principle of animal-environment mutuality. Behavior and Philosophy.

[CR16] Coulter J, Sharrock W (2007). Brain, mind, and Human Behavior in Contemporary Cognitive Science: Critical assessments of the philosophy of psychology.

[CR17] Damasio A (1994). Descartes’ error: Emotion, reason, and the human brain.

[CR18] Damasio A (1999). The feeling of what happens: Body, emotion and the making of consciousness.

[CR19] Derksen M (2010). Realism, relativism, and evolutionary psychology. Theory & Psychology.

[CR21] Fassin D (2009). Another politics of life is possible. Theory Culture & Society.

[CR22] Finlay S, Roth C, Zimsen T, Bridson TL, Sarnyai Z, McDermott B (2022). Adverse childhood experiences and allostatic load: A systematic review. Neuroscience & Biobehavioral Reviews.

[CR67] Fitzgerald, D., & Callard, F. (2015). Social Science and Neuroscience beyond Interdisciplinarity: Experimental Entanglements. *Theory, Culture & Society*, *32*(1), 3–32. 10.1177/0263276414537319 10.1177/0263276414537319 PMC4425296 25972621

[CR23] Fletcher JR, Birk RH (2022). The conundrum of the psychological interface: On the problems of bridging the biological and the social. History of the Human Sciences.

[CR24] Frances A (2013). Saving normal.

[CR25] Friston K (2012). The history of the future of the bayesian brain. Neuroimage.

[CR26] Fuchs T (2018). Ecology of the brain.

[CR27] Gannik D (1995). Situational disease. Family Practice.

[CR28] Gannik D (2005). Social sygdomsteori: Et situationelt perspektiv.

[CR29] García-Gutiérrez, M. S., Navarrete, F., Sala, F., Gasparyan, A., Austrich-Olivares, A., & Manzanares, J. (2020). Biomarkers in Psychiatry: Concept, Definition, Types and Relevance to the Clinical Reality. *Frontiers in Psychiatry, 15;11*, 432. 10.3389/fpsyt.2020.00432 PMC7243207 32499729

[CR30] Gibson JJ (1986). The Ecological Approach to Visual Perception. (first published 1979).

[CR31] Gier NF (1981). Wittgenstein and Phenomenology: A comparative study of the later Wittgenstein, Husserl, Heidegger and Merleau-Ponty.

[CR32] Greenwood JD (2015). A conceptual history of psychology: Exploring the tangled web.

[CR33] Horwitz AV, Wakefield JC (2007). The loss of sadness: How Psychiatry transformed normal sorrow into depressive disorder.

[CR34] Ingold T, Fabian AC (1998). The evolution of society. Evolution: Society, Science and the Universe.

[CR35] Ingold T, Rose H, Rose S (2000). Evolving skills. Alas, poor Darwin: Arguments against evolutionary psychology.

[CR36] Ingold T (2011). Being Alive: Essays on Movement, knowledge and description.

[CR37] Ingold T (2015). The life of lines.

[CR38] Ingold T, Palsson G (2013). Biosocial Becomings: Integrating Social and Biological Anthropology.

[CR39] James, W. (1890/1950). *The Principles of Psychology*.Dover Publications, Inc.New York.

[CR40] Kawamura S (1959). The process of sub-culture propagation among japanese macaques. Primates.

[CR41] Krieger N (2005). Embodiment: A conceptual glossary for epidemiology. Journal of Epidemiology & Community Health.

[CR42] Laland K, Matthews B, Feldman MW (2016). An introduction to niche construction theory. Evolutionary Ecology.

[CR43] Latour B (2005). Reassembling the Social.

[CR44] Mammen J, Mironenko I (2015). Activity theories and the ontology of psychology: Learning from danish and russian Experiences. Integrative Psychological and Behavioral Science.

[CR45] Martin E (2021). Experiments of the mind: From the cognitive psychology lab to the World of Facebook and Twitter.

[CR46] Meloni M (2014). How Biology Became Social, and what it means for Social Theory. Sociological Review.

[CR47] Mol A (2002). The body multiple: Ontology in Medical Practice.

[CR48] Niewöhner J, Lock M (2018). Situating local biologies: Anthropological perspectives on Environment/Human entanglements. BioSocieties.

[CR49] Ostenfeld EN (2018). Ancient greek psychology and the modern mind-body debate.

[CR50] Panofsky A (2014). Misbehaving Science: Controversy and the development of Behavior Genetics.

[CR51] Piaget J (1971). Biology and Knowledge.

[CR52] Rose N (1996). Inventing our selves: Psychology, power, and Personhood.

[CR55] Rose N, Abi-Rached JM (2013). Neuro: The New Brain Sciences and the management of the mind.

[CR56] Rosenberg CE (2007). Our Present complaint: American Medicine, then and now.

[CR57] Singh I, Rose N (2009). Biomarkers in psychiatry. Nature.

[CR58] Slife BD, Burchfield C, Hedges D (2010). Interpreting the “biologization” of psychology. The Journal of Mind and Behavior.

[CR59] Smith SE (2020). Is evolutionary psychology possible?. Biological Theory.

[CR60] Spear JH (2007). Prominent schools or other active specialties? A fresh look at some trends in psychology. Review of General Psychology.

[CR61] Sterelny K (2012). The Evolved apprentice: How Evolution made humans unique.

[CR62] Stotz K (2014). Extended evolutionary psychology: The importance of transgenerational developmental plasticity. Frontiers in Psychology.

[CR63] Whitehead PM (2017). Goldstein’s self-actualization: A biosemiotic view. The Humanistic Psychologist.

[CR64] Wilson EA (2004). Psychosomatic: Feminism and the neurological body.

[CR65] Withagen R, van Wermeskerken M (2010). The role of affordances in the evolutionary process reconsidered: A niche construction perspective. Theory & Psychology.

[CR66] Wittgenstein L (1953). Philosophical investigations.

